# Obstructive Pseudotumor of Tuberculosis in a Young Woman: A Rare Presentation

**DOI:** 10.1155/2014/914253

**Published:** 2014-09-02

**Authors:** Seyyed Reza Fatemi, M. Ghobakhlou, L. Alizadeh

**Affiliations:** Research Center for Gastroenterology and Liver Diseases, Taleghani Hospital, Shahid Beheshti University of Medical Sciences, Tabnak Street, Evin, Tehran, Iran

## Abstract

Retroperitoneal pseudotumor is an extremely rare presentation of extrapulmonary tuberculosis. The diagnosis of this paucibacillary disease is difficult which is usually misdiagnosed as a malignant tumor. High index of suspicion is required for early diagnosis and treatment of retroperitoneal pseudotumor which can affect prognosis of this disease. Because of its rarity and difficult diagnosis, we report an 18-year-old immunocompetent girl who presented with abdominal pain and vomiting. Upper endoscopy showed an exudative mass between the second and third parts of duodenum. Abdominal computed tomography (CT) revealed a large retroperitoneal mass with extension into small bowel. Exploratory laparotomy and histopathological examination of tissue showed calcified granuloma. Ziehl-Neelsen staining and PCR confirmed the tuberculosis. The patient was successfully treated with standard antituberculosis therapy.

## 1. Introduction

*M. tuberculosis* or nontuberculous mycobacterial infections can present as a rare and distinct entity called mycobacterial pseudotumors. M. pseudotumors affect immunosuppressed patients with or without AIDS [1]. One of the most common sites for extrathoracic presentation of tuberculosis is abdomen [2]. Tuberculosis may involve any abdominal organ [1]. Pseudotumor of abdomen is a rare and distinct clinical entity. In fact extrapulmonary tuberculosis is rare and retroperitoneal mass is extremely rare manifestation of extrathoracic tuberculosis [1, 3–6]. Inflammatory pseudotumor occurs because of diverse processes including infective or reactive/reparative processes or low grade mesenchymal malignancy [7]. The diagnosis of abdominal pseudotumor is often delayed because of the different clinical manifestations that lead to serious complications. The diagnosis of typical forms is easy, but some forms are misleading and can lead to an incorrect diagnosis [4]. The abdominal mass is similar to a tumor and is often misdiagnosed as a malignant tumor which is diagnosed after surgery [2, 8–11]; as a result, patients may be referred to oncologist and surgeon, postponing the initiation of antituberculosis therapy. Abdominal pseudotumor of tuberculosis cannot be usually diagnosed by culture and even polymerase chain reaction (PCR) and definite diagnosis is made through surgery [10–12]. To facilitate timely antituberculosis therapy, the prompt diagnosis by clinicians is necessary.

We report a young female with abdominal pain and vomiting who was referred to our institution with a primary diagnosis of malignancy.

## 2. Case Report

The patient was an 18-year-old girl who presented with periumbilical abdominal pain, nausea, vomiting, fatigue, and powerless four months prior to admission that these symptoms had gradually increased. She had five kg weight loss. She had no history of any diseases.

On physical examination, she appeared ill. Vital signs were normal. Abdomen was soft; a periumbilical mass was detected in deep palpation; no hepatomegaly, splenomegaly, or lymphadenopathy was detected.

The hemoglobin level was 10.2 g/dL. The leukocyte count was 5400/mm^3^ (neutrophils: 57%, lymphocytes: 41%) and the platelet count was 291000/mm^3^. The aspartate aminotransferase was 32 U/L and alanine aminotransferase was 28 U/L. The total bilirubin was 1.3 mg/dL and direct bilirubin was 0.5 mg/dL. The alkaline phosphatase was 250 U/L. The serum lactate dehydrogenase (LDH) was 110 IU/L. The blood urea nitrogen (BUN) was 25 mg/dL and the creatinine was 0.5 mg/dL. The erythrocyte sedimentation rate was 30.

**Figure 1:**
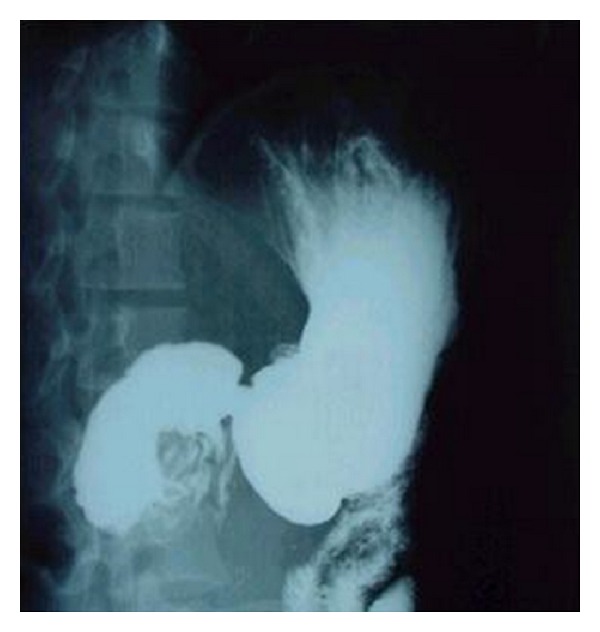
Upper GI series showing cut-off area between the second and third parts of duodenum.

**Figure 2:**
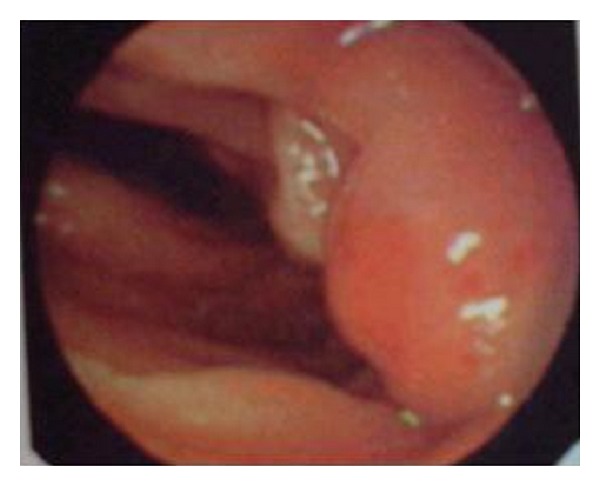
Upper endoscopy showing an obstructive and exudative mass between the second and third parts of duodenum.

**Figure 3:**
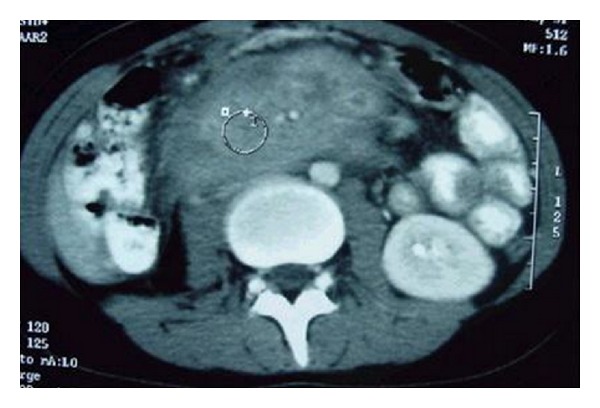
Abdominal computed tomography showing an 8 × 5 ×  7 cm heterogeneous lesion with indefinite borders invading to the proximal part of small bowel in retroperitoneal space.

Small bowel barium study revealed a cut-off area between the second and third parts of duodenum (Figure 1). Upper gastrointestinal endoscopy was performed and showed an obstructive and exudative mass at the same region (Figure 2). Endoscopic biopsy showed only nonspecific inflammation. Chest X-ray was normal. Abdominal computed tomography (CT) showed an 8 × 5 × 7 cm heterogeneous mass with indefinite borders in retroperitoneal space at the level of kidneys; some free fluid was seen in pelvic area (Figure 3).

Our primary differential diagnosis was retroperitoneal lymphoma, GIST, or desmoid tumor. Patient underwent surgery. Laparotomy was performed and a large fragile mass was seen with adhesion to transverse mesocolon and small intestinal tension; significant vascular involvement was seen; pancreas, liver, biliary tree, spleen, and the ovaries appeared normal; multiple biopsies were taken and gastrojejunostomy was performed for obstruction.

**Figure 4:**
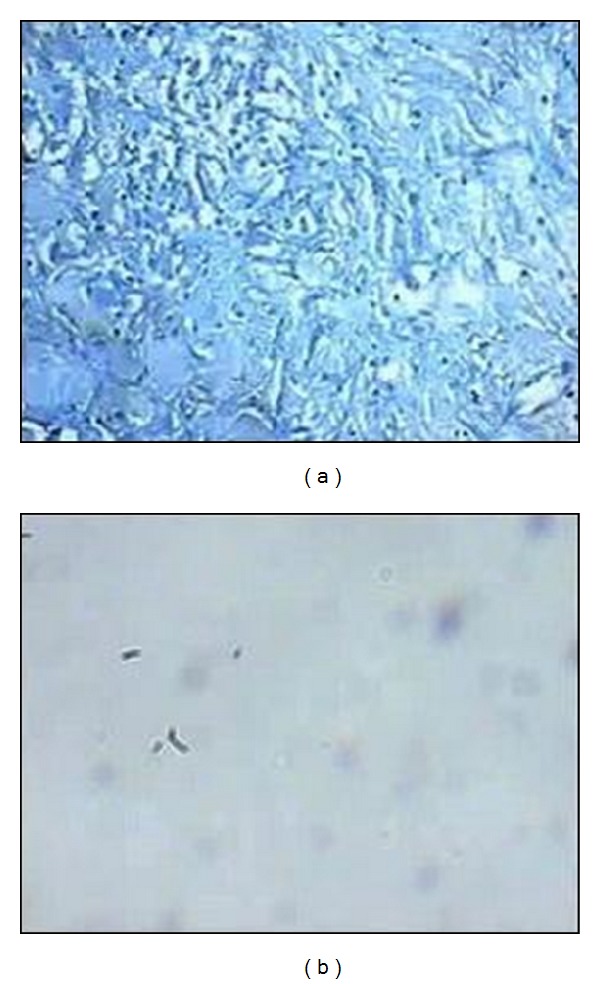
Microscopic pathologic examination showing reactive tissue with necroinflammatory infiltration, langerhans and giant cells, and calcified granuloma (a); Ziehl-Neelsen staining showing acid-fast bacilli (b).

Microscopic pathologic examination revealed reactive tissue with necroinflammatory infiltration, langerhans cells, and calcified granuloma (Figure 4(a)). Ziehl-Neelsen staining showed acid-fast bacilli (Figure 4(b)). Polymerase chain reaction (PCR) was positive for mycobacterium tuberculosis.

Quadruple therapy for tuberculosis with isoniazid, rifampin, pyrazinamide, and ethambutol was prescribed for a two-month initial phase of treatment followed by a four-month continuation phase of isoniazid and rifampin. In patient's follow-up, general condition was good and no problem was reported.

## 3. Discussion

The pseudotumors of tuberculosis have mostly been reported in mediastinum [3, 6, 7]; sometimes, tuberculosis can present as a mass lesion at liver hilum that is diagnosed by surgery [6]. In spite of imaging advances, diagnosis of this pseudotumor is finally made by surgery or sampling [8, 12]. Abdomen is considered to be one of the most prevalent sites for manifestation of tuberculosis [4]. Any abdominal organ may be involved in localization of tuberculosis, but symptoms of abdominal pseudotumors are not specific and diagnosis can often be missed and mimic malignancy. Because of their polymorphic presentation physicians should be aware of this distinct entity and apply all modern diagnostic procedures [8].

Retroperitoneum is an extremely rare region of involvement of tuberculosis and can be misdiagnosed as a neoplastic tumor in imaging study [2, 3, 7]. Abdominal mass and retroperitoneal tuberculosis which have increased in recent years only contribute to 1% of clinical manifestations of disease [5].

The differential diagnosis of retroperitoneal mass is lymphoma, leiomyosarcoma, GIST, desmoid tumor, Wegener's granulomatosis, inflammatory pseudotumor, and tuberculosis [13–17].

Tuberculosis may be detected by chest X-ray (33%) and abdominal CT (88%), but there were no pathognomonic criteria and the diagnosis is ultimately based on histopathological and microbiological confirmation [18–20]. In the past, surgery was necessary to sampling, but, today's, tissue sample can be obtained with minimal invasion, under image-guidance [20]. The polymerase chain reaction is a rapid and reliable test even for contaminated specimens, with a good sensitivity (76.4%) and high specificity (99.8%) [21, 22]. However, diagnosis of this paucibacillary disease is very difficult that leads to delay in treatment. In this condition, even diagnostic assays, such as culture and PCR, may provide negative results [3, 22].

This patient's manifestation was an obstructive lesion in small intestine and further evaluation revealed a large retroperitoneal tumor with extension to the small intestine.

Our primary impression was lymphoma or leiomyosarcoma but surgery and pathologic examination revealed that the retroperitoneal tuberculosis has been manifested as a pseudotumor.

This form of tuberculosis usually manifests in setting of underlying tumors and immune deficiency [19]. Clinicians should be aware of this unusual manifestation of tuberculosis which affects immunosuppressed patients but interestingly our patient had intact immune system. This unique response to* M. tuberculosis* may be because of individual immunological response of the patient [1, 4]. Iran is considered as an endemic area for tuberculosis; therefore, reactivation of latent infection should be considered as possible diagnosis, even in an immunocompetent host. With appropriate diagnosis and treatment, pseudotumor is resolved during treatment. In conclusion, retroperitoneal pseudotumor should be kept in mind in the differential diagnosis of abdominal masses, particularly in endemic regions.
